# Using satellite data to monitor land-use land-cover change in North-eastern Latvia

**DOI:** 10.1186/2193-1801-3-61

**Published:** 2014-01-30

**Authors:** Simon Foteck Fonji, Gregory N Taff

**Affiliations:** Department of Earth Sciences, University of Memphis, 001 Johnson Hall, Memphis, TN 38152 USA; Norwegian Forest and Landscape Institute, Tromso, Norway

**Keywords:** Land-use and land-cover change, Remote sensing, LANDSAT, Land-cover change detection, Latvia, GIS

## Abstract

Land-use and land-cover change (LULCC), especially those caused by human activities, is one of the most important components of global environmental change (Jessen 3^rd^ edition: 1-526 2005). In this study the effects of geographic and demographic factors on LULCC are analyzed in northeastern Latvia using official estimates from census and vital statistics data, and using remotely sensed satellite imagery (Landsat Thematic Mapper) acquired from 1992 and 2007. The remote sensing images, elevation data, in-situ ground truth and ground control data (using GPS), census and vital statistics data were processed, integrated, and analyzed in a geographic information system (GIS). Changes in six categories of land-use and land-cover (wetland, water, agriculture, forest, bare field and urban/suburban) were studied to determine their relationship to demographic and geographic factors between 1992 and 2007. Supervised classifications were performed on the Landsat images. Analysis of land cover change based on “change-to” categories between the 1992 and 2007 images revealed that changes to forest were the most common type of change (17.1% of pixels), followed by changes to agriculture (8.6%) and the fewest were changes to urban/suburban (0.8%). Integration of population data and land-cover change data revealed key findings: areas near to roads underwent more LULCC and areas far away from Riga underwent less LULCC. Range in elevation was positively correlated with all LULCC categories. Population density was found to be associated with most LULCC categories but the direction of effect was scale dependent. This paper shows how socio-demographic data can be integrated with satellite image data and cartographic data to analyze drivers of LULCC at multiple spatial scales.

## Introduction

Land-use change and land-cover change (LULCC) are terms often used interchangeably but the two have different meanings. Land cover describes the natural and anthropogenic features that can be observed on the Earth's surface. Examples include deciduous forests, wetlands, developed/built areas, grasslands, water, etc. Land use, by contrast, describes activities that take place on the land and represent the current use of property. Examples include residential homes, shopping centers, tree nurseries, state parks, reservoirs, etc. In land change science, land cover and land use are often studied in conjunction with each other, especially in studies involving remote sensing, because satellite imagery and aerial photography can identify land-cover, however inferring land-use often requires more knowledge of the study region, and therefore some compromise is often made between identifying the variable of interest (land use) and the related proxy (land cover).

LULCC, especially those caused by human activities, is one of the most important components of global environmental change (Jensen 2005). Land cover change also plays an important role in local and regional environmental change. LULCC is local and place-specific, and collectively these changes add up to global environmental change. These changes in turn affect other components of the earth - atmosphere system, often with adverse consequences such as biodiversity loss, desertification, and climate change.

There are many ways to monitor or detect land cover change over time. In the past scientists used field data and aerial photographs to map LULCC over smaller areas. As the size of the area of study gets bigger, these methods become very costly and time consuming. Remote sensing via satellite imagery is an excellent tool to study LULCC because images can cover large geographic extents and have a high temporal coverage. Remote sensing is also used to investigate historical LULCC and also provide data (e.g. ground truth) in areas that are inaccessible. The major disadvantages of remote sensing include: the inability of many sensors to obtain data and information through cloud cover, distinct phenomena can be confused if they look the same to the sensor, the resolution of the satellite imagery may be too coarse for detailed mapping and for distinguishing small contrasting areas and very high-resolution satellite imagery are very expensive. Despite these disadvantages, remotely sensed satellite data have been used to identify changes in a variety of aquatic and terrestrial environments including coastal, agriculture, forested, and urban areas (Berlanga and Ruiz 2002). This is particularly true for remote regions, which are often inaccessible and therefore not easy to obtain the needed data using traditional methods (Roberts et al. 2003; Cingolani et al. 2004). LULCC researchers often use remotely sensed data to provide information on resource inventory and land use, and to identify, monitor and quantify changing patterns in the landscape. Population change and distribution is a significant driver of LULCC in many regions of the world. With the emergence of GIS in the past two decades, census data have been merged with biophysical data to better understand the drivers of LULCC at local, regional and global scales. For example, the combination of satellite classification and census data has been used to assess quality of life (Lo and Faber 1997), predict favorable wolf habitat in northern Wisconsin (Mladenoff et al. 1995), assess the effect of population change on forest cover in Ghana between 1990 and 2010 (Codjoe 2004), predict future LULCC in Bindura district-Zimbabwe (Kamusoko et al. 2009), understand the socioeconomic driver s of change in the Ecuadorian Amazon (Mena, Bilsborrow, and McClain 2006), understand change in agricultural activities in the Brazilian Amazon (Cardille and Foley 2003), comprehend relationships between land-cover and housing density in Wisconsin (Radeloff et al. 2000), and to study deforestation in the Brazilian Amazon (Wood and Skole 1998). Dewan et al. (2009) studied land-use and land-cover changes and urban expansion in Greater Dhaka, Bangladesh, between 1975 and 2003 using satellite images and socioeconomic data. The present research integrated land cover change data (based on Landsat TM images from 1992 and 2007) and demographic data (from the Latvian demographic censuses and inter-census estimates based on vital statistics data for 1992 and 2007) at the level of rural parishes (in Latvian: pagasti) and counties (in Latvian: rajoni) in a GIS to determine associations between LULCC and demographic factors (population density and population growth between 1992 and 2007).

Analysis of land-cover change across thirty six countries in Europe shows a slowing in the annual rate of changes in land-cover types from 2000-2006 compared to the period 1990-2000 (EEA 2010). The EEA report also indicates that land-use specialization (urbanization, agricultural intensification and abandonment plus natural afforestation) is still a very strong trend and is expected to continue into the future, depending on many interactive drivers. Land marginalization and intensification are trends in Europe that have an impact on the European landscape that promote homogenization and polarization of rural landscape (Antrop 2006). EEA report 2010 shows that forest creation and management was the largest land-cover change while arable land, permanent crop, pasture, open spaces and wetlands continue to decline in area. When landscapes change, living organisms must adapt to the changes, depending on whether the changes are sudden or over a long period of time. Thus it is important to measure out how quickly the landscape has changed, i.e. rate of change (Antrop 2000).

Eastern Europe experienced a period of rapid and radical changes of its political, institutional, demographic, and socioeconomic structures after the fall of the Iron Curtain in 1989 and the breakdown of the Soviet Union in 1991 which triggered widespread land use change, most notably the abandonment of vast areas of cropland in Latvia and other countries in the region (Taff et al. 2010). According to Kuemmerle et al. (2009), political and socio-economic changes in central and eastern Europe in the 20^th^ and 21^st^ century have accentuated trends in land-use change.

Latvia has experienced several major transformations in land use over the course of the 20^th^ century (Nikodemus et al. 2005) and farming systems in Latvia have changed substantially in relation to changing socio-economic context with heavy impacts on the landscape (Peneze 2009). During the Soviet period (1945-1990) large areas were used for agriculture in the plains, while in marginal and hilly areas, forestry became the dominant land-use (Peneze et al. 2009). After Latvia gained its independence from the Soviet Union in 1991, the government decided to reinstate lands to private owners. This period also saw a change from a socialist economy to a capitalist economy which drove Latvia away from an agriculture-dominated economy, and many farms were abandoned (Taff et al. 2010). From 2004 until the first half of 2008, Latvia had the most rapidly developing economy in the European Union, with the GDP growth reaching 12.2% in 2006 (Eurostat 2010); then in 2009 the Latvian economy suffered a severe setback, one of the worst among the European Union Member States (GDP of-18%) forcing substantial outmigration to find employment. There has been a net population decrease and also a drift, especially of younger people to the cities, resulting in an ageing and decreasing rural population. Studies regarding the impact of migration in land-use change especially in Europe are very limited (Lopez et al. 2006). In rural Latvia this effect is very pronounced as many farms have been abandoned due to outmigration from rural areas to cities. Land privatization and the increase in economic wealth during the 2000’s drove substantial changes in land-use, particularly agricultural abandonment (Taff et al. 2010) and the recent development of urban sprawl in Latvia and many East European countries (Geyer 2011).

This research uses statistical analyses to address the drivers of LULCC, using data from remote sensing images, topographic maps, and census data. Two summer dates of LANDSAT Thematic Mapper images from June 4, 1992 and August 9, 2007 (Path/Row 186/020) were processed, classified and analyzed, and an accuracy assessment was performed using ERDAS and ArcGIS. Post-classification change detection was used to determine LULCC between the two dates. Census data were then analyzed in conjunction with the LULCC results to understand associations between landscape changes and demographic and geographic variables. The specific research question this paper addresses is: how are local geographic and demographic factors associated with LULCC in Latvia in the time frame since its independence? Land-use change categories analyzed in this study are agricultural abandonment and reforestation, new agricultural development, and urban/suburban development, and forest clearing. While some research has shown that population increase rates do not always mirror urban/suburban development rates (Eetvelde and Antrop 2004), an increase in population is generally expected to accompany increases in urban and suburban development. Research has shown that agricultural abandonment in rural areas is often, though not always, associated with outmigration from a region (Gellrich and Zimmermann 2007).

## Materials and methods

### Study area

**Figure 1:**
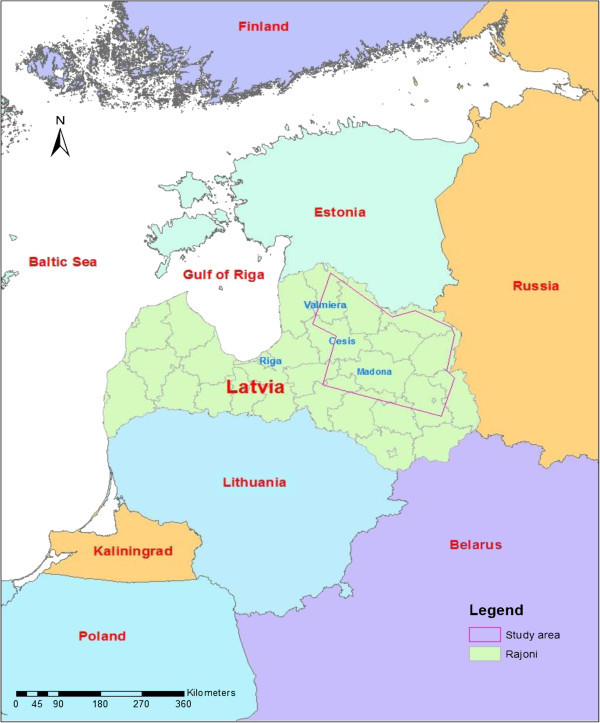
**Study area north-eastern Latvia.**

The study area is located in northeastern Latvia which covers an area of 18,193.99 sq. km and includes 199 pagasti (parishes) in 14 rajoni (districts). Latvia lies on the Baltic coast, in the northern part of Eastern Europe. Latvia is one of three Baltic States situated on the east side of the Baltic Sea, the others being Estonia (to the north) and Lithuania (to the south). Latvia also borders Russia and Belarus to the east (Figure 1). The total land area of Latvia is 64.6 thousand km^2^ and the terrain is mostly low plain, with the majority of the territory between 40-200 meters above sea level (Eberhards 1984). The climate is wet with moderate winters for this latitude. The average amount of precipitation is 750-850 mm annually of which 500 mm falls in the warm period and the average temperature is 16.5°C (Ruskule et al. 2012). The vegetation period usually lasts for 180-200 days (Normunds 1993). Rural landscape in this region is characterized by matured forests, secondary forest, meadows, farmland, abandoned farmlands, lakes, rivers, hills, plains, villages and dispersed rural homesteads (Bunkse 2000).

Latvia had a population of 2.07 million people in 2011, according to the 2011 census (Latvian Central Statistical Bureau website 2013). After the middle of 2008, Latvia experienced a particularly severe economic setback along with the world economic crisis, which forced substantial outmigration for work opportunities. The increased outmigration, in addition to one of the lowest birth rates in the world, caused an increase in annual population loss (Zvidrins 1998 Central Statistical Bureau of Latvia 2008). Most of Latvia’s export is from wood and agricultural products (Eurostat 2010). Therefore, maintaining the health of Latvia’s rural ecosystems is important to the economy of Latvia. During the summer of 2011, the author, along with a research team, collected field data of ground control points and ground truth of relevant land-cover types in the study area, which lies in the country’s north-east, in the Vidzeme region, and parts of the Latgale and Zemgale regions (Figure 1). This region was chosen because of the wide variety of demographic changes in the region, including areas of out-migration and some of the few areas within Latvia experiencing in-migration, and it encompasses some of the fastest developing cities in the country, such as Valmiera, Sigulda, and Cesis.

### Data

In this study two Landsat Thematic Mapper (TM) images (path 186, row 20) with a spatial resolution of 30x30 meters were acquired from June 4, 1992 and August 9, 2007. Summer images were chosen to best distinguish the spectral signatures of the different land-cover types, and near-anniversary dates were chosen for consistency between the two time points. Cloud cover in the southwest of the image required the creation of a subset of the image without that portion. Layered boundary data such as roads, rivers, protected areas, and municipal boundary data were collected from SIA Environtech (Environtech LTM). In addition, municipal boundaries data were obtained from 2004 of rajoni and pagasti (two out of the fourteen rajoni had their entire areas within the study area and the rest were partially enclosed within the study area and 55 out of 199 pagasti had only part of their area within the study area while the rest had their entire area within the study area). Only areas within the study area were considered for analyses. Population data from the Latvian Central Statistical Bureau (taken from Latvian Censuses and updated via estimates from vital statistics data) are also used in this study. Only areas with municipal boundaries within the study area were considered for analysis. A 90 m resolution Digital Elevation Model (DEM) was downloaded from the (DIVA-GIS 2010). The DEM was resampled to 30 m × 30 m resolution using the nearest neighbor technique to match the resolution of the LANDSAT images. Slope and aspect were derived from the DEM using spatial analyst in ArcGIS. However, it should be noted that, there are errors inherent in DEM data that affects the overall quality of the data and derivatives (slope and aspect). The various data types used in this study are fully described in Table 1.

**Table 1:** **Data description**

	Class types determined from reference source								
		Water	Wetland	Forest	Agric	Bare field	Urban/suburban	Totals	User’s accuracy
Class types determined from classified map.	Water	50	0	0	0	0	0	50	100%
	Wetland	0	36	0	0	0	0	36	100%
	Forest	0	0	48	3	5	1	57	84.21%
	Agric	0	5	2	45	5	6	63	71.43%
	Bare field	0	1	0	1	35	1	38	92.11%
	Urban/suburban	0	0	0	1	0	40	41	97.56%
	Totals	50	42	50	50	45	48	285	
Producer’s accuracy		100%	86%	96%	90%	77.78%	83.33%	Total accuracy	89.12%
Overall Kappa statistics = 0.869									

### Geometric correction

Ground control points were collected during field work in the summer of 2010 to georectify the 2007 image. Ground control points consisted primarily of major road intersections that have not changed over the study period, based on Google Earth images of 2007 and earlier dates, and paper base maps. The1992 image was then co-registered to the 2007 image in ERDAS 9.3. Both images were registered to a common Universal Traverse Mercator (UTM) projection. The 2007 image was geometrically corrected using a second-order polynomial and re-sampled using the nearest neighbor technique. The total root mean square (RMS) error for the 2007 image was approximately 2.5 meters, which is quite good for this study given that the RMS error should be at most half the size of the pixel (15 m) (Campbell 2002).

### Land cover classification scheme

This study used per-pixel supervised classifications which group satellite image pixels with the same or similar spectral reflectance features into the same information categories (Campbell 2002). In addition to using relevant LULC classes, all classes of interest must be carefully selected and defined to successfully classify remotely sensed data into land-cover (or land-use) information (Gong and Howarth 1992).

Six information classes of interest to this study were chosen from the National Land Cover Database for the Conterminous United States (Homer et al. 2007) and also correspond to the CORINE land cover classes (http://www.eea.europa.eu/publications/COR0-landcover) and supported by field observations based on dominant land-cover types in Latvia. CORINE land cover 2000 (CLC2000) also provides useful information about land cover types in 24 European countries including Latvia and information about land-cover changes can be obtained from CLC data (Feranec et al. 2010). In this study we decided to create our own custom data sets using a much finer resolution (30 m × 30 m) based on the resolution of Landsat TM. The goal of this study is focused on identifying and analyzing the causes of LULCC associated with three main processes: forest clearing, forest growth (including agricultural abandonment), and development. Note that in this study, forest refers to forest standing only. The six classes used were urban/suburban (built-up), agriculture, bare field/barren (including clear cut areas), forest, water, and wetlands. Bare soil was used as an additional class for classification of the Landsat images, but for land-use change analyses the agriculture and bare soil classes were merged into an agriculture land-use class, since bare soil in Latvia generally represents agriculture – fallow or recently harvested.

**Figure 2:**
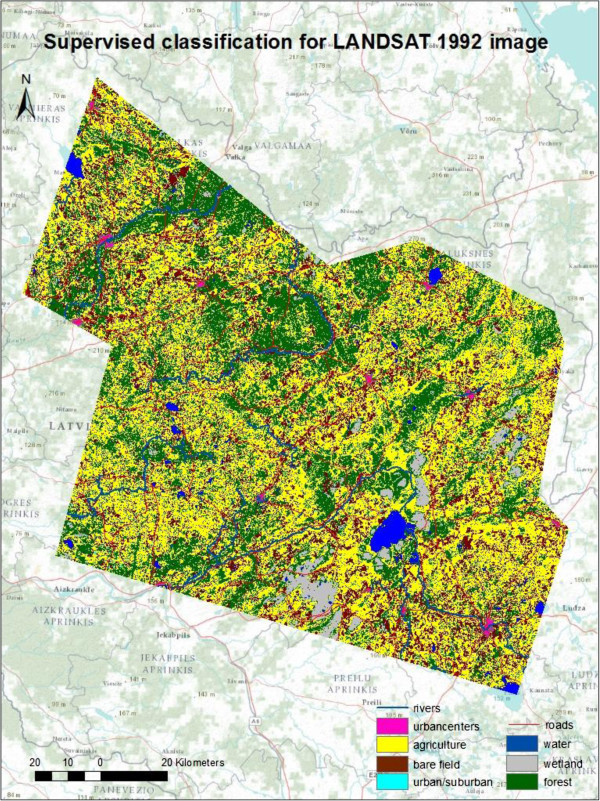
**Maps of supervised classification 1992.**

**Figure 3:**
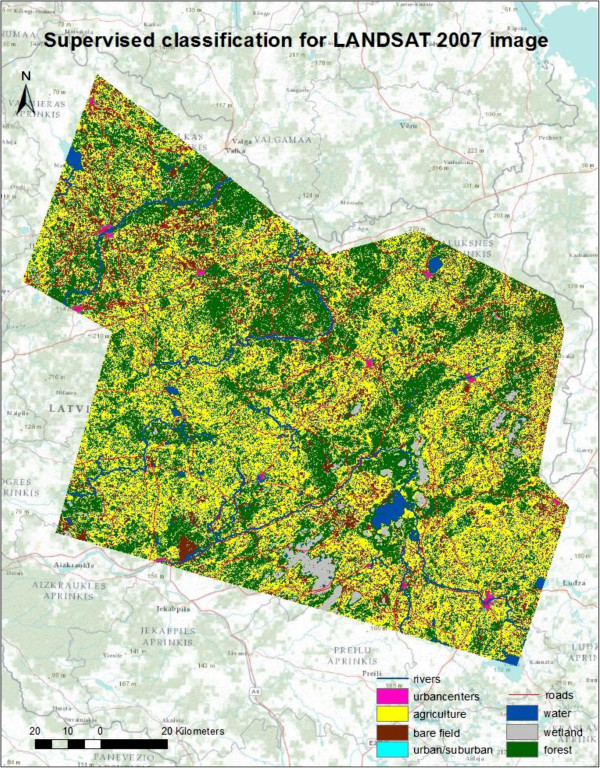
**Maps of supervised classification 2007.**

Maximum likelihood supervised classifications were performed in ERDAS IMAGINE 9.3 on the 1992 and 2007 Landsat images. For each class, 10 ground-truth polygons were digitized based on air photos and visual analysis of locations on Google Maps and the image itself. In accordance with Jensen (2005), each polygon contained at least 50 pixels except in a few cases where a high proportion of land cover patches in the study area contained fewer than 50 pixels (for the suburban and water classes). To improve classification, training polygons with confusing spectral signatures were discarded and new ones created based on a visual analysis of the locations in Google Maps and on the image itself, and these were added to the existing samples and the maximum likelihood algorithm was run again. Pixels throughout the classified image were visually compared with the raw image and Google Maps to determine classification accuracy. This process was repeated until the observable errors in the classification were negligible. Figures 2 and 3 show the final outputs of the supervised classification, which consists of two classified maps of northeast Latvia, in 1992 and 2007.

### Accuracy assessment

An accuracy assessment was performed for the 2007 image, because of good available ground truth at this date. Ground-truth for the 1992 image was not possible to obtain, but the same classification methodology was used for both images. For the 2007 image, three hundred pixels were selected randomly (50 pixels from each class) and checked for accuracy using fieldwork ground truth data when available, aerial photographs and GoogleMaps. 15 cases were unclear from visual inspection of the imagery what type of land-cover was present, so these points were discarded. The standard summaries are reported for the accuracy assessment: the error matrix, the overall accuracy and the Kappa coefficient (Congalton 1991). Error matrices quantitatively compare the relationship between the classified maps and reference data. The overall accuracy for the 2007 classified map based on the supervised classification was 89.12% which is considered good, and it is above the limit set by USGS guideline (85%). Because the over all accuracy assessment tends to overestimate the actual performance, a more useful representation of performance is the Kappa coefficient (Cohen 1960). The Kappa coefficient for the supervised 2007 image was 0.869 which means that 86.9% of the classification is better than a random classification. This is considered good because a Kappa value above 80% is considered to have a strong agreement (Ramita et al. 2009). Table 2 shows the results for the accuracy assessment for the supervised classification of the 2007 image.

**Table 2:** **Classification accuracy assessment of 2007 image using error matrix**

Accuracy assement for supervised classification									
	Class types determined from reference source								User’s accuracy
		Water	Wetland	Forest	Agriculture	Bare field	Urban/suburban	Totals	
Class types determined from classified map	Water	50	0	0	0	0	0	50	100.00%
	Wetland	0	36	0	0	0	0	36	100.00%
	Forest	0	0	48	3	5	1	57	84.21%
	Agric	0	5	2	45	5	6	63	71.43%
	Bare field	0	1	0	1	35	1	38	92.11%
	Urban/suburban	0	0	0	1	0	40	41	97.56%
	Totals	50	42	50	50	45	48	285	
**Producer’s accuracy**		100%	86%	96.00%	90.00%	77.78%	83.33%	Total accuracy :	89.12%
Overall Kappa statistics = 0.869									

## Results

### Land-use change analysis

**Figure 4:**
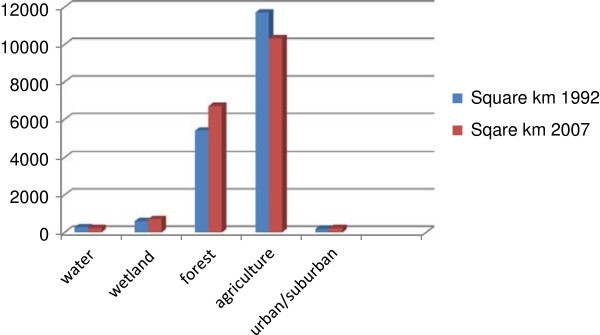
**Land-cover classes and area represented by each class in square kilometers for 1992 and 2007.**

Bare soil and agriculture were recoded as one class because in this study area most of the bare soil land-cover class generally represented land-uses that were either pastures or agricultural fields without crops at that moment (e.g., recently harvested or fallow). A total of five classes were produced for each of the two images (water, wetlands, forest, agriculture, and urban/suburban). The 1992 image and the 2007 image were compared in terms of the total area of each land cover category. As seen in Table 3 and Figure 4, the classes that have increased in area include wetland, forest, and urban, while water and agriculture have decreased over the same time period. The substantial amount of decrease in water is primarily due to damming of lakes in selected parts of the study site especially in the southeast of the study area where there is a large reduction in water cover from 1992 to 2007. Agriculture areas have decreased primarily due to farm abandonment and development (construction of buildings, roads, and houses). The reduced percentage of agriculture can be attributed to farm abandonment that started primarily in 1991 after Latvia gained independence from the former Soviet Union and the agricultural sector became less profitable due to the shift to a capitalist economy and the breakup of farms into smaller plots (through land restitution) . Increase in forest may also be due to farm abandonment that resulted in the conversion of many agricultural fields into young forest. The persistence beyond the early post-Soviet transition years of increase in forest cover follows the forest transition theory (Rudel et al. 2005), which hypothesizes that forest cover decreases with early development levels of a society for resource extraction, but then increases with an even higher level of development due to a change in sector focus of the economy and an increased demand for maintenance of green space. This period also saw a population shift within Latvia to bigger cities in search of jobs which led to a recent development of urban sprawl (increased buildings, and roads at the urban periphery and beyond). Development can be seen throughout the study area, especially in cities and suburban areas of Valmiera, Cesis, Sigulda, and Madona.

**Table 3:** **Land-cover classes and area represented by each class in square kilometers for 1992 and 2007**

Land cover classes	Area in square km in 1992	Area in square km in 2007
Water	274.2633	228.4434
Wetland	598.1832	705.3327
Forest	5433.4458	6716.5758
Agriculture	8575.9794	7617.7575
Bare field	3125.7864	2700.9117
Urban/suburban	186.3315	224.9685

**Figure 5:**
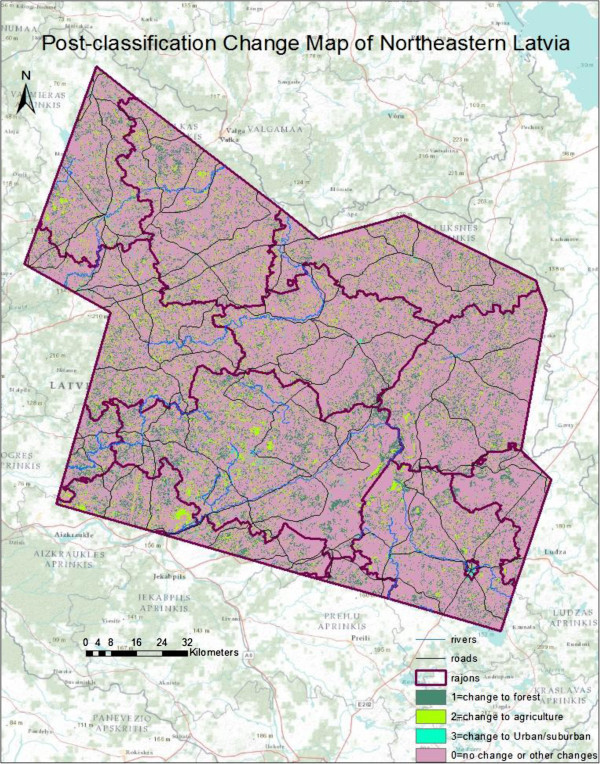
**Post-classification "change-to"map.**

A pixel-level “from-to” change analysis was then run with five classes and the result was a change map with twenty five classes. In order to reduce the number of classes in the change map, “no-change” classes and “change” classes that were considered unimportant to the study objectives were classified as irrelevant (with a value of 0), all the classes that *changed to* forest were classified as 1, all classes that *changed to* agriculture were classified as 2, and all classes that *changed to* urban were classified as 3 (Figure 5).

**Figure 6:**
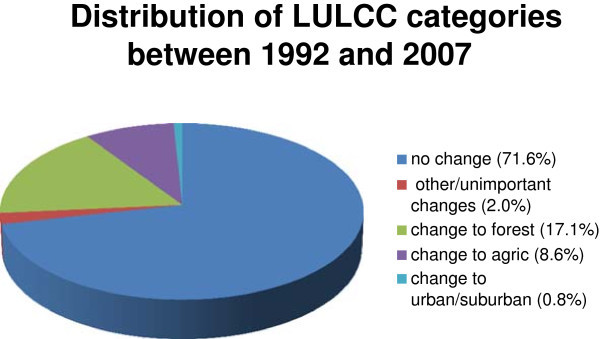
**Distribution of LULCC categories between 1992 and 2007 of Northeastern Latvia.**

**Figure 7:**
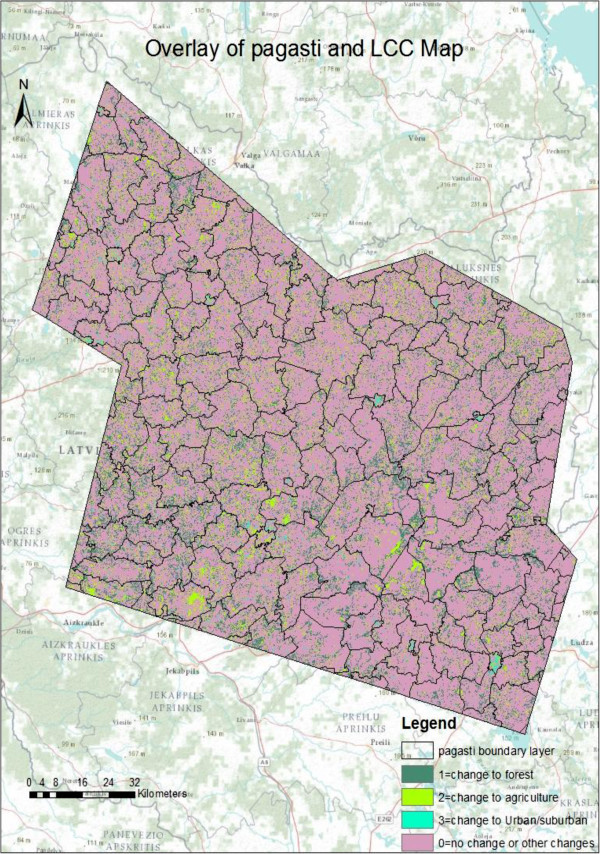
**Overlaying of the pagasti layer of Northeastern Latvia with land cover change map of Landsat TM image.**

**Figure 8:**
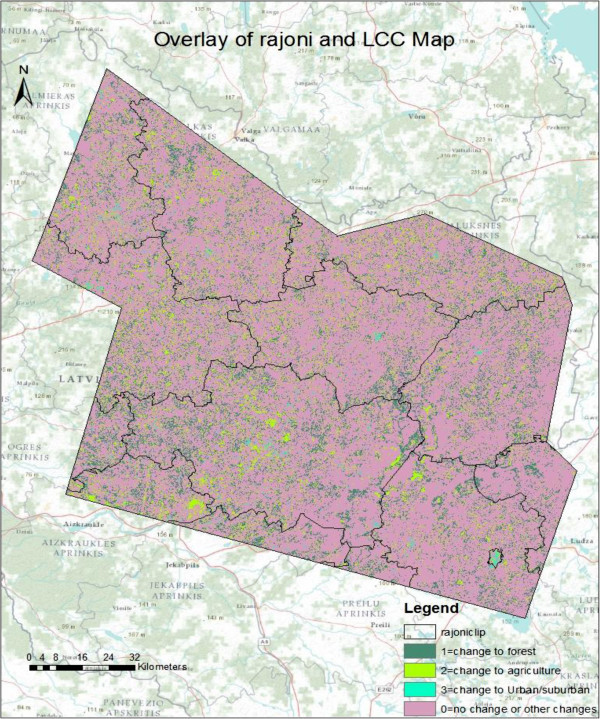
**Overlaying of the rajoni layer of Northeastern Latvia with land cover change map of Landsat TM images.**

Among change classes, land cover types that changed to forest were most common (17.1%), followed by classes that changed to agriculture (8.6%), and finally classes that changed to urban (0.8%). The “No-change” class (71.6%) combined with changes considered unimportant (2%) for this study comprised 73.6% of the pixels (Figure 6). Finally, the LULCC map was overlaid with population data at the level of pagasti (municipality) and rajoni (similar to US counties) as shown in Figures 7 and 8.

LULCC classes were then extracted and aggregated at these two spatial units of analysis, namely rural pagasti (excluding big cities) and rajoni (including all major cities). Demographic data from January of 1993 and 2008 were used to best represent years for the corresponding dates of the Landsat images from one year prior. The percent of each LULCC class was calculated in each of the pagasti and rajoni, and correlations were run between the proportion of each LULCC class and 1) socio-demographic factors (population density in 2008, and percent population growth rate between 1993 and 2008) and 2) geographic and topographic factors. The results are shown in Table 4. The results show that, at the pagasti level, percent of pagasti area within 1 km from major roads (excluding dirt roads) and range in elevation are positively correlated with all three types of land cover change variables (i.e. percent change to agriculture, forest and urban/subur ban). Therefore, it seems that the more infrastructures a pagast has (e.g. roads) the more rural development occurs (Creightney 1993). According to Chomitz and Gray (1996) rural roads not only promote economic development but they also facilitate deforestation. The significance with range in elevation is interesting; as a low range in elevation has been found in other studies to be associated with land-use changes, mostly occurring in coastal areas and in areas having low slope values (Selcuk 2008). In this study site, the higher ranges in elevation tend to follow the Gauja River Valley, along which fall the towns of Sigulda, Cesis and Valmiera; in addition, the areas near the slopes of this valley are popular tourist destinations. These two drivers (nearby cities and park recreation areas) likely promote development, reforestation (especially within the nearby Gauja National Park), and apparently agriculture as well (perhaps due to road accessibility or proximity to these rural towns). The positive relationship between range in elevation and the other types of land-use change may seem surprising (since development is not likely to occur at high elevations or on high slopes). But the range in elevation in the study area varies between 0-270 m and most of the highest points are located near big cities and close to the Gauja River. Development in such areas may reflect a desire to be near these natural areas and cities. A similar study that revealed a positive correlation between range in elevation and land use change was reported by Turner et al. (1996). The percent of rajoni within 10 km of an urban area was calculated, and found to be significantly negatively correlated with the change to forest. This means rajoni with more areas within 10 km from urban centers experienced less changes to forest and is likely due to the higher value of lands near urban centers, both for agriculture and conversion to urban cover; therefore, less agricultural land was converted to forest in these areas. Percent population growth rate was not significantly correlated with percent change to forest and agriculture but it was positively correlated with percent change to urban at the level of pagasti. At the rajoni scale, percent population growth rate was not significantly correlated with any of the land cover change variables. Percent of pagasti areas within 10 km from urban centers and percent population growth rates were highly correlated with a change to urban/suburban development. These results are as expected: development occurs to accommodate population growth near cities. Also, the more increase in population a pagasti has, the more urban development will occur to accommodate them. Factors that significantly negatively correlated with some land-use changes at the pagasti scale were population density in 2008 and proximity to the capitol, Riga. New agricultural lands and forestry lands were found in pagasti with low population densities. At the rajoni scale, there was a significant positive correlation between population density and change to forest, which is surprising, as it shows agricultural abandonment in high population areas; this may be due to the fact that most of the highest population rajoni have substantial area inside Gauja National Park, where forest regrowth was promoted through government policy (Taff 2005). Distance to Riga was found to be negatively correlated with all land-cover change variables, and significant with some of them–on both the rajoni and the pagasti scales. Increased distance to Riga was associated with decreases in land-cover changes. New agriculture, forestry, and urbanization are happening more near Riga than far away. A similar study carried out by Jat et al. (2008), showed that urbanization in Ajmer city was driven by population concentration in the area. The result regarding urbanization near Riga is as expected, and the results regarding increased forestry and agriculture nearer to Riga may have to do with distance to market (cost of carrying goods to Riga) or because people are engaging in farming/forestry activities part time, while they or family members have other employment in or near Riga. In this study, protected areas were also considered, defined here as areas designated as national parks/forest reserves under legislation primarily aiming at nature conservation (EEA Report, 2012). Protected areas have been particularly efficient in avoiding deforestation processes, which is considered the major threat to biodiversity (Burner et al. 2001). Administrative units inside protected areas were expected to show less change in LULCC than those outside protected areas, however the percent protected areas within rajoni did not have significant correlation with any of the land cover change variables, though it was significantly positively correlated with a change in urban/suburban at the pagasti scale. This likely due to the fact that Gauja National Park, which covers many of the pagasti in the study area, became a national park in 1973, so very little development occurred in the park compared to outside the Park until Latvian independence in 1991. Then throughout the 1990’s, land restitution occurred and over 80% of the Park was privatized. This land privatization was the likely impetus for new development at a higher rate compared with land outside the Park, where development was allowed during the late Soviet era (1970’s - 1991). Also, range in elevation was not found to be significantly correlated with any of the land cover variables at the rajoni scale.

**Table 4:** Correlation of socio-demographic and geographic variables and LULCC at the level of pagasti and rajoni; (*) significant correlation (p < 0.05), (**) significant correlation (p < 0.01)

	Socio demographic/ geographic factors	% Change to agriculture	% Change to forest	% Change to urban/suburban
	% Pagasti area within 10 km of urban centers	-0.029	-0.082	.175*
	P-values	0.72	0.302	0.027
	% Pagasti area within 1 km from roads	.213^**^	.162^*^	.209^**^
	P-values	0.007	0.041	0.008
	Population density 2008	-.160^*^	-.236^**^	-0.068
	P-values	0.043	0.003	0.394
	% Population growth 1993-2008	0.054	-0.076	.215**
Pagasti	P-values	0.5	0.338	0.007
	% Protected area within pagasti	0.144	0.096	.180*
	P-values	0.07	0.228	0.023
	Proximity to Riga in Km	-.286**	-0.086	-.550**
	P-values	0	0.28	0
	Range in elevation	.360^**^	.280^**^	.304^**^
	P-values	0	0	0
Rajoni	% Rajoni area within 10 km of urban centers	-0.289	-.533^*^	-0.078
	P-values	0.316	0.05	0.791
	% Rajoni area within 1 km from roads	-0.297	-0.34	-0.138
	P-values	0.303	0.234	0.639
	% Population growth 1993-2008	0.318	0.472	0.503
	P-values	0.268	0.088	0.066
	Population density 2008	-0.032	.645^*^	0.526
	P-values	0.913	0.013	0.053
	% Protected areas within rajoni	0.002	-0.275	0.247
	P-values	0.994	0.341	0.395
	Proximity to Riga	-0.523	-0.452	-.727^**^
	P-values	0.055	0.104	0.003
	Range in elevation	-0.181	0.043	0.231
	P-values	0.535	0.883	0.427

## Conclusion

LULCC monitoring in northeastern Latvia was achieved using post-classification change detection. The results demonstrate how remote sensing can be used to assess, monitor and quantify LULCC in large areas where traditional methods (such as field observation) may not be possible. The results from this study revealed changes in some landscape patterns in northeastern Latvia in the post-Soviet period. According to Vanwambeke et al. (2012), the structure of the landscape in Vidzeme (part of the study area) has changed significantly over the past 20 years in relation to deep restructuring of the political and economic systems in Latvia. The most significant land cover change experienced in the study area was increase in forest cover. The percent of forest cover loss between 1992 and 2007 was 8.3% and percent of forest cover gain over the same time period was 17.1%. Out of the 17.1%, 15.71% resulted from the conversion of agriculture to forest cover. Much of this change can be attributed to reforestation resulting from agricultural abandonment, as is common throughout the former Soviet Union since 1991 (Taff et al. 2010). Reforestation on abandoned agricultural lands was most common in low population density pagasti, i.e. 70.2% of land cover that changed to forest occurred in pagasti that had less than ten people per square kilometers. The increase in urban/suburban cover occurred mostly around big cities and in high population density pagasti. Between 1992 and 2007, the percent of agriculture loss was 17.5% and percent of agriculture gain was 8.6%.

Among the socio-demographic and geographic variables used to explain land cover change in this region, the most contributing factors with positive correlation to all three land cover change variables were percent of pagasti/rajoni area within 1 km from roads, and range in elevation. Distance to Riga had a negative correlation with all the land cover change variables at both the pagasti and rajoni scales. Some of the factors that had a significant correlation at the pagasti scale (e.g. elevation) had no significant correlation at the rajoni scale. In general, based on comparison between the pagasti and rajoni correlations, it appears that the rajoni scale is too broad to catch the appropriate processes with regards to some of the demographic and geographic variables.

Latvia has been experiencing serious depopulation and low fertility levels (Zvidrins 1998; European Environment agency 2010) since independence in 1991. The demographic statistics show that most of the rural pagasti (147 out of 159) have experience a decrease in their population between 1993 and 2008. This research shows that regions of rural depopulation in Latvia are coupled with substantial agricultural abandonment. While an increase in forest cover may increase the country’s capacity to sequester carbon, provide clean air, prevent erosion, and address some other environmental issues, it is worthwhile to note that decrease in agricultural land in this region can lead to decrease in biodiversity, loss in cultural landscape and some tourist opportunities (Taff 2005), and loss in food production in the region, which can lead to reliance on foreign imports and a trade imbalance. The food production index (covers food crops that are considered edible and that contain nutrients) in Latvia has dropped from 22 in 1992 to 138 in 2009 (World Bank Indicators 2010). These results on patterns of change in agriculture and associated variables can help policy makers to address key relevant variables associated with loss of agriculture, which can help them address food security, cultural landscape maintenance, and employment issues–the rate of unemployment in Latvia in 1992 was 3.178% and in 2010 was 18.965% (International Monetary Fund 2011). Of the three land cover change types in this study, the lowest percentage of land cover (1.02%) was converted to urban/suburban, and most of this development took place near cities. Results of this study therefore suggest the need to monitor increases in urban/suburban cover near cities, and to plan suburban development to avoid many problems typically associated with sprawl. The general land-use change trends found in this study, and the variables found to be associated with these land-use changes, can be useful to policy makers beyond the study site in the northeast to all of Latvia and beyond regarding land-use planning based on geographic and population characteristics to address issues of land-use, economic development, and food security. There are several factors that could contribute to the improvement of the analyses in this research. For instance, in this LULCC analysis, finer scale socio-demographic and economic variables were not used because of their lack of availability at the city scale, but such fine-scale variables likely had significant influence on land-use change. Future studies should research the association of socio-demographic and geographic variables at the city scale and incorporate more economic variables.
